# Survivin and caspases serum protein levels and survivin variants mRNA expression in sepsis

**DOI:** 10.1038/s41598-020-78208-2

**Published:** 2021-01-13

**Authors:** Marianna Miliaraki, Panagiotis Briassoulis, Stavroula Ilia, Aikaterini Polonifi, Marina Mantzourani, Efrossini Briassouli, Konstantinos Vardas, Serafim Nanas, Aikaterini Pistiki, Maria Theodorakopoulou, Theonymfi Tavladaki, Anna Maria Spanaki, Eumorfia Kondili, Helen Dimitriou, Sotirios Tsiodras, Dimitrios Georgopoulos, Apostolos Armaganidis, George Daikos, George Briassoulis

**Affiliations:** 1 Pediatric Intensive Care Unit, Medical School, University of Crete, Heraklion, Crete Greece; 2 First Department of Internal Medicine - Propaedeutic, National and Kapodistrian University of Athens, Athens, Greece; 3 First Critical Care Department, Evangelismos University Hospital, National and Kapodistrian University of Athens, Athens, Greece; 4 4th Department of Internal Medicine, Attikon University Hospital, National and Kapodistrian University of Athens, Athens, Greece; 5 2nd Department of Critical Care, Attikon University Hospital, Medical School, National and Kapodistrian University of Athens, Athens, Greece; 6 Intensive Care Unit, Medical School, University of Crete, Heraklion, Crete Greece; 7 Division of Mother and Child Health, Medical School, University of Crete, Heraklion, Crete Greece; 8 Postgraduate Program “Emergencies and Intensive Care in Children Adolescents and Young Adults”, Medical School, University of Crete, Heraklion, Crete Greece

**Keywords:** Biomarkers, Medical research, Pathogenesis

## Abstract

Sepsis is a dysregulated host response to infection related to devastating outcomes. Recently, interest has been shifted towards apoptotic and antiapoptotic pathobiology. Apoptosis is executed through the activation of caspases regulated by a number of antiapoptotic proteins, such as survivin. The survivin and caspases’ responses to sepsis have not yet been elucidated. This is a multicenter prospective observational study concerning patients with sepsis (n = 107) compared to patients with traumatic systemic inflammatory response syndrome (SIRS) (n = 75) and to healthy controls (n = 89). The expression of survivin was quantified through real-time quantitative polymerase chain reaction for the different survivin splice variants (wild type-WT, ΔEx3, 2B, 3B) in peripheral blood leukocytes. The apoptotic or antiapoptotic tendency was specified by measuring survivin-WT, caspase-3, and -9 serum protein concentrations through enzyme-linked immunosorbent assay. The survivin-WT, -2B, -ΔΕx3 mRNA, survivin protein, and caspases showed an escalated increase in SIRS and sepsis, whereas survivin-3B was repressed in sepsis (*p* < 0.05). Survivin correlated with IL-8 and caspase-9 (*p* < 0.01). For discriminating sepsis, caspase-9 achieved the best receiver operating characteristic curve (AUROC) of 0.95. In predicting mortality, caspase-9 and survivin protein achieved an AUROC of 0.70. In conclusion, specific apoptotic and antiapoptotic pathways might represent attractive targets for future research in sepsis.

## Introduction

Sepsis is a complex clinical condition, defined as a dysregulated host response to infection, leading to multi-organ failure and death^1,2^. Since a dysregulated apoptosis seems to contribute to the development of multi-organ failure, rising future strategies in sepsis might be based on cell survival studies^3,4^.

Caspases are cysteine proteases that cleave their substrates on the C-terminal side of aspartate, leading to DNA fragmentation, membrane blebbing, phosphatidylserine exposure at the cell surface, and apoptotic vesicles formation^5^. Crucial biological functions of caspases are linked to cell death in apoptosis and pyroptosis^6,7^, as well as to non-cell death functions in inflammation, dendrite trimming, cell differentiation and migration^8^, following two distinct pathways^9^. The extrinsic apoptotic pathway is activated through the binding of a ligand to a death receptor, which leads to the activation of caspase-8, the major mediator of this apoptotic cascade^6^. The intrinsic (mitochondrial) pathway is activated through various cellular stresses that lead to the release of apoptotic factors sequestered by the mitochondria, such as cytochrome c, resulting in the activation of caspase-9 and loss of the mitochondrial membrane integrity^10^. Caspase-3 is the terminal executioner protease in both pathways of apoptosis, leading to disruption of the nuclear envelope and breakdown of genomic DNA, via cleavage of structural proteins. Low levels of caspase-3 activity are necessary for critical developmental processes in several cell types^11^.

Inhibitors of apoptosis proteins (IAPs), including the survivin protein, seem to restrain the downstream components of caspase-activation pathways and play important roles in regulating the progress of apoptosis, but their role in sepsis has not been elucidated so far. Survivin is an evolutionarily conserved eukaryotic protein (BIRC5) that is expressed in actively proliferating cells, playing a crucial role in cell division by inhibiting apoptosis and regulating the process of mitosis in embryonic and cancer cells^12^. Survivin’s upregulation in malignancies has extensively been reported^13^, while accumulated evidence shows that it exerts cell-protection in non-malignant conditions as well^14^. Apoptotic deficiency seems to play an important role in the survival of lymphocytes, leading to autoimmunity. Given that survivin is essential for mitosis, in maintaining homeostasis of the immune system, and able to inhibit apoptosis^15^, piled evidence favors the involvement of survivin dysregulation in the development of inflammatory disorders^16^.

The importance of apoptotic/antiapoptotic balance is revealed by complex regulatory cascades involving multiple factors, such as the CD40 ligand or members of the Bcl-2 family, which suppress apoptosis, whereas Fas ligand, tumor necrosis factor α (TNFα) and cytokines shift the balance toward pro-apoptotic signaling^17^. This delicate balance might be significantly impaired in sepsis and may represent a new target for future scientific exploration. Certain alterations, in favor of apoptotic cascades, may play an important role in the development of septic shock and sepsis-related mortality, while investigators have speculated that prevention of apoptosis may be important in sepsis in order to prevent immune suppression^18^.

The current study was designed to test the hypothesis that a significant apoptotic/antiapoptotic imbalance characterizes septic ICU patients, especially those at greater risk of death. The main goal is to determine the main differences regarding the early-onset cumulative apoptotic (caspases) and the antiapoptotic (survivin protein) status, amongst patients with septic shock or sepsis compared to patients with non-infectious (traumatic) systemic inflammatory response syndrome (SIRS) or healthy controls. We hypothesized that sepsis might induce caspase-3, -9, and survivin variants expression, a finding that could open up a new path in exploring the pathophysiology in sepsis.

## Results

### Patients

A total number of 328 ICU patients were initially screened. Seventy-eight individuals (78) not fulfilling the inclusion criteria, or who denied consent, were excluded, while 68 were excluded at the propensity data matching analysis. One hundred-seven (107) patients fulfilling the criteria of Sepsis-3 and 75 patients with traumatic SIRS, consecutively admitted to the ICUs, were enrolled in the study within 24 h from admission. The control groups included 89 healthy volunteers. Patient demographic and clinical characteristics are presented in Table 1. Mortality, disease severity scores, heart rate, CRP, and lactate were higher in sepsis compared to traumatic SIRS (*p* < 0.05).

**Table 1:** Patients demographics and clinical characteristics.

Characteristic	Control (n = 89)	SIRS (n = 75)	Sepsis (n = 107)	*p*^#^
Age (years), mean ± SE	51.9 ± 1.5	49.15 ± 1.9	54.9 ± 1.6	0.076
Sex (Female/Male), (%)	39/50 (44/56)	26/49 (35/65)	44/63 (41/59)	0.478
ICU LOS (days), mean ± SE		25 ± 4.71	24 ± 2.67	0.863
Mortality ICU, n (%)		9 (12)	42 (38)^†^	0.001
APACHE score, mean ± SE		15 ± 0.73	22.4 ± 0.79^†^	0.001
SOFA score, mean ± SE		8.3 ± 0.3	10 ± 0.3^†^	0.001
SAPS score, mean ± SE		48.8 ± 1.5	71.5 ± 1.7^†^	0.001
WBC × 10^3^ (cells/μL), median (IQR)		12.6 (9.5–16.7)	13.9 (8.6–20)	0.232
Lactate (mg/dl), median (IQR)		4.3 (2–12.5)	4.7 (1.95–23)	0.575
CRP (mg/dl), median (IQR)		9.5 (4.2–28.5)	25.6 (13–89)^†^	0.001
Procalcitonin (ng/ml), median (IQR)		0.77 (0.44–1.82)	5 (1.14–28.8))^†^	0.011
IL-6 (pg/ml), median (IQR)	3.5 (1.25–14.8)**	77 (19.5–157)*	86 (27.6–391)	0.001
IL-10 (ng/ml), median (IQR)	3.7 (0.5–11.3)**	10.2 (0.01–23.2)*	16.7 (5.3–64.7)^†^	0.001
IL-8 (pg/ml), mean ± SE	82.7 ± 14.2	84.3 ± 27.5	139 ± 24.4	0.109

^#^Differences among groups (*ANOVA, Kruskal–Wallis, x*^2^* test*, as appropriate): Post hoc differences: *control-SIRS, **control-sepsis, **†**SIRS-sepsis.

*SIRS* systematic inflammatory response syndrome, *IQR* interquartile ratio, *ICU* intensive care unit, *LOS* length of stay, *WBC* white blood cells, *CRP* C-reacting protein, *IL* Interleukin.

### Survivin transcriptional expression and survivin/caspases serum concentrations

**Figure 1:**
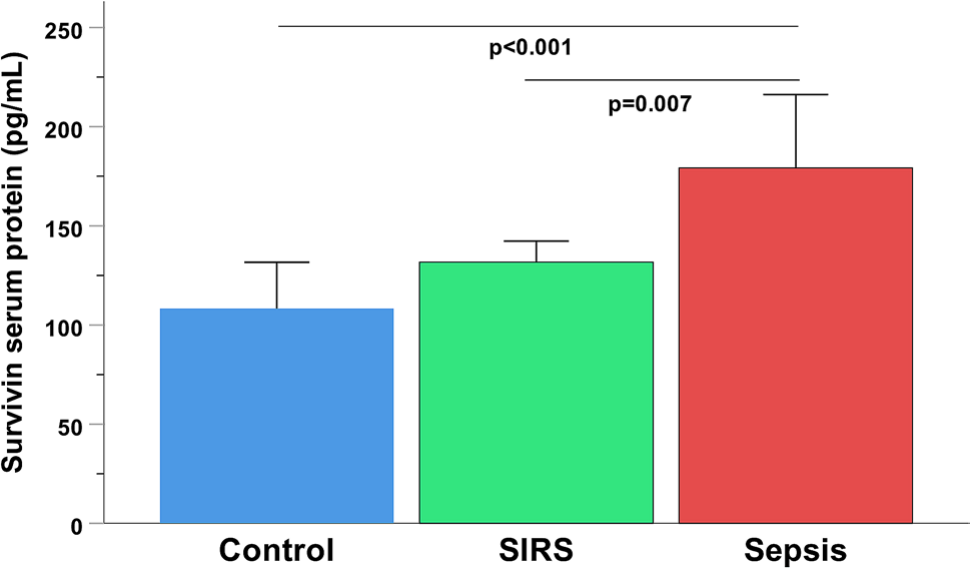
Serum levels of survivin-WT (isoform 1 or alpha) in septic patients in comparison to patients with non-infectious (trauma) systemic inflammatory response syndrome (SIRS) and healthy controls. Bars represent Medians, error bars 95% Confidence Intervals. Connectors indicate significantly higher levels in sepsis.

**Figure 2:**
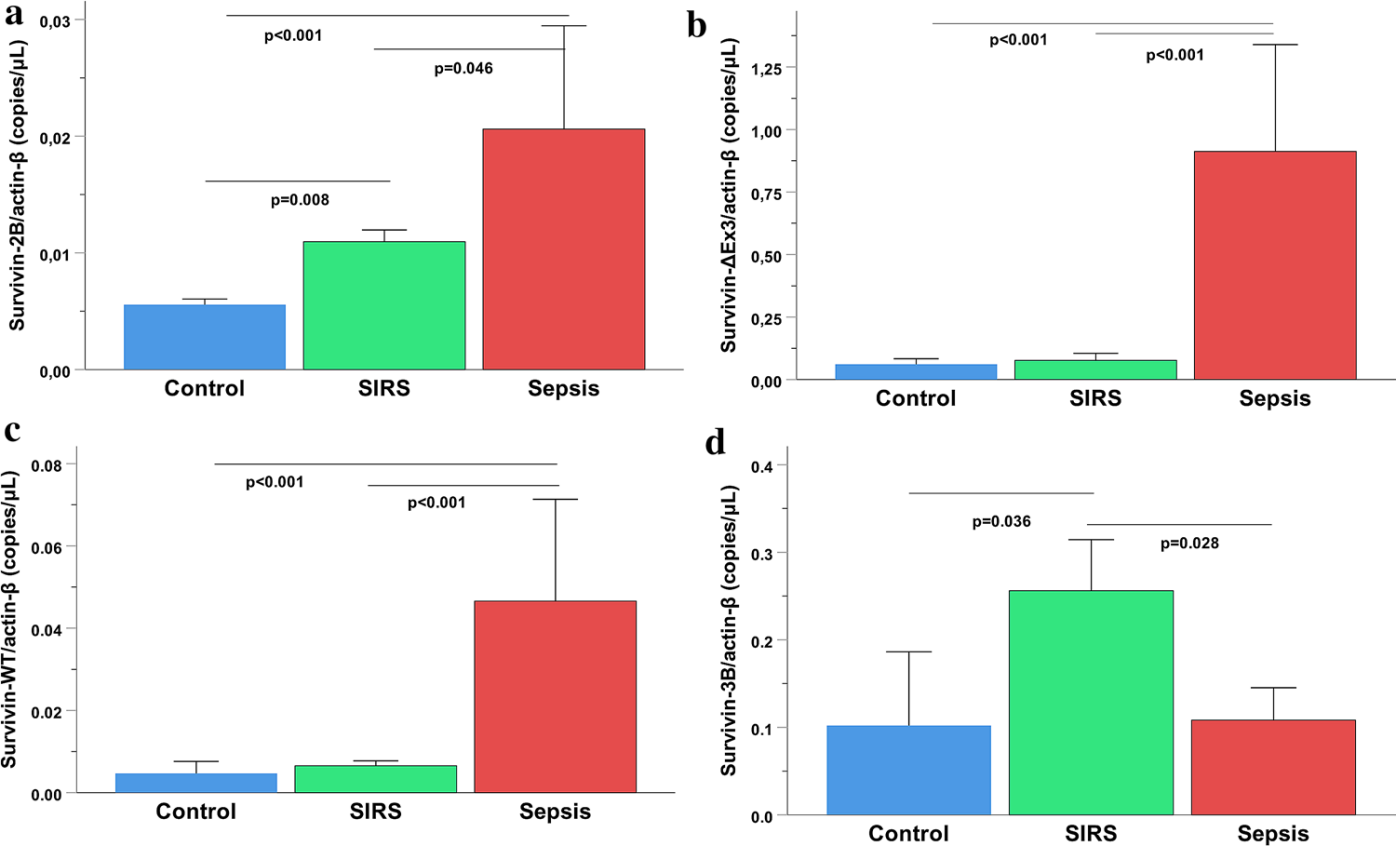
Gene expression levels of survivin isoforms: (**a**) Survivin-2B; (**b**) Survivin-ΔΕx3; (**c**) Survivin-WT; and (**d**) Survivin-3B in the three study groups. Bars represent Medians, error bars 95% Confidence Intervals. Connectors indicate significant difference between septic and non-septic critically ill or healthy individuals.

**Figure 3:**
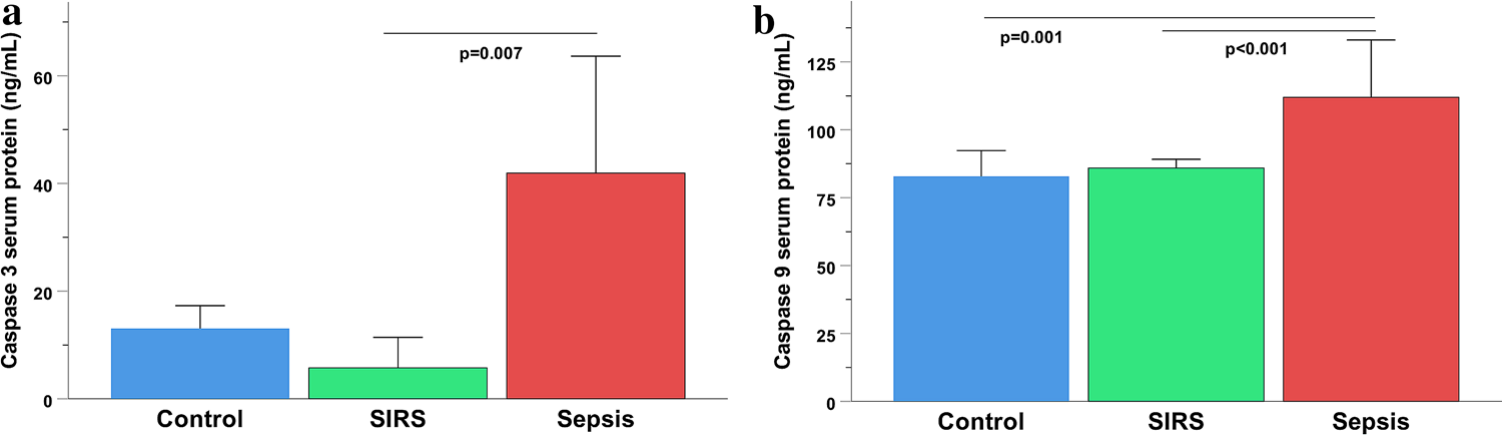
Serum levels of total caspases enzymatic concentrations, including pro-caspases, the cleaved subunits, and the active heterodimers: (**a**) Caspase-3 and (**b**) Caspase-9 in septic patients compared to SIRS and healthy controls. Bars represent Medians, error bars 95% Confidence Intervals. Connectors indicate statistical significant difference between septic and non-septic critically ill or healthy individuals.

Serum levels of the survivin wild-type protein were elevated in septic patients compared to traumatic SIRS and control individuals (*p* < 0.001) (Fig. 1). Survivin-WT and splice variant -ΔΕx3 were the dominant isoforms, showing the highest levels of expression in patients’ samples. The -2B, -ΔΕx3 and -WT transcriptional levels were increased in sepsis compared to controls and/or SIRS (*p* < 0.001), whereas survivin-3B was repressed in sepsis compared to SIRS (*p* = 0.028) (Fig. 2a–d). A concurrent upregulation of total (pro-caspase, active and cleaved) caspase-3 and -9 enzymatic concentration was observed in the septic group, compared to traumatic SIRS and control individuals (*p* < 0.01) (Fig. 3a,b).

### Relations: associations

Caspase-9 was significantly related to survivin protein (rs = 0.34, *p* < 0.01) and WT variant (rs = -0.33, *p* < 0.03), APACHE II, SOFA, SAPS III scores (rs = 0.63, *p* < 0.001), and procalcitonin (PCT) (rs = 0.76, *p* < 0.01), but not to CRP or lactate. Caspase-3 presented better correlation with the survivin-ΔΕx3 isoform (rs = 0.52, *p* < 0.001), whereas survivin correlated with IL-8 (rs = 0.41, *p* < 0.05). In a logistic regression model, only caspase-9 was independently associated with sepsis among critically ill patients (Exp(B) = 1.2, 95%CI = 1.09–1.4, *p* < 0.04).

### Discriminating sepsis

**Figure 4:**
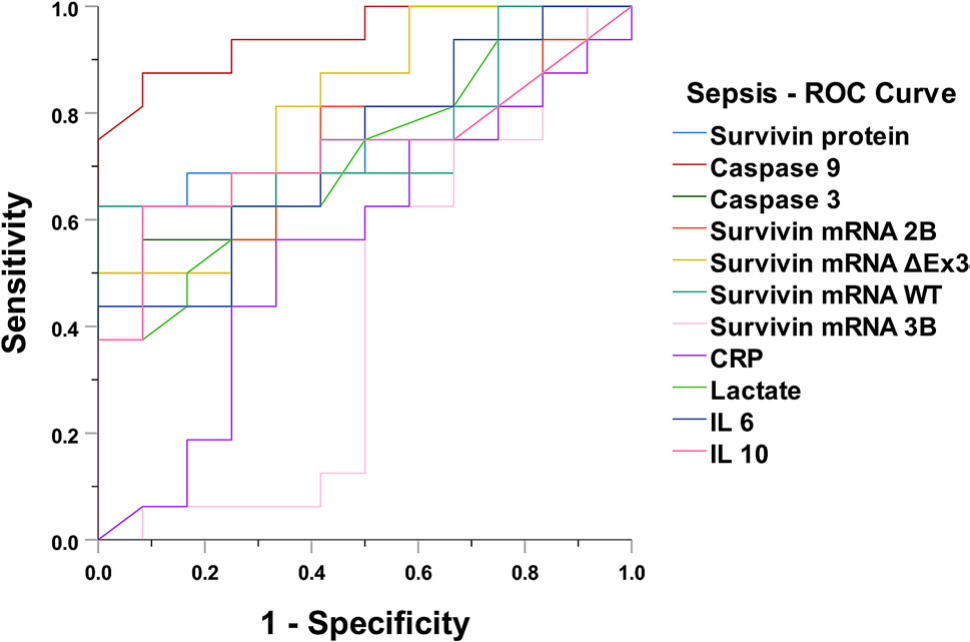
ROC curve for discriminating sepsis. Caspase-9 achieved the best receiver operating characteristic curve (AUROC) of 0.95 (95% CI 0.87–0.99, *p* < 0.001).

For discriminating sepsis, caspase-9 achieved the best receiver operating characteristic curve (AUROC) of 0.95 (95% CI 0.87–0.99, *p* < 0.001), along with survivin-ΔEx3 = 0.81 (95% CI 0.57–0.97, *p* < 0.005), survivin-WT = 0.76 (95% CI 0.57–0.94, *p* < 0.03), and survivin protein = 0.76 (95% CI 0.58–0.95, *p* < 0.02) (Fig. 4), with CRP exhibiting the worst AUROC = 0.59 (95% CI 0.37–0.83, *p* < 0.388) (Appendix Table A1).

### Predicting mortality

**Figure 5:**
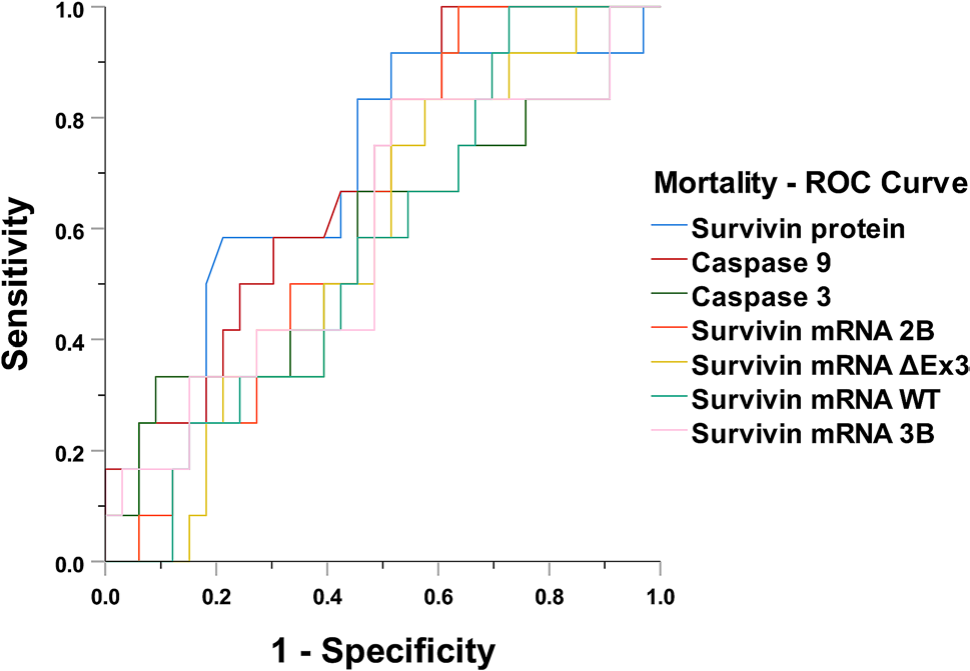
ROC curve for predicting mortality. Caspase-9 and survivin protein achieved the best receiver operating characteristic curve—AUROC of 0.70 (95% CI 0.52–0.88, *p* < 0.05).

Survivin protein serum concentration (*p* < 0.001), as well as splice variants -WT and -2B were elevated in the mortality group of patients, compared to sepsis survivors (*p* < 0.02). Caspases also showed a weak but statistically non-significant tendency to be upregulated in the mortality group (Table 2). In predicting mortality among critically ill patients, caspase-9 and survivin protein achieved an AUROC of 0.70 (95% CI 0.52–0.88, *p* < 0.05) (Fig. 5, Appendix Table B1).

**Table 2:** Survivin’s expression and caspases serum levels related to mortality.

Laboratory Assay	Survival	Mortality	*p*^*#*^
Survivin-2B (copies/μl), median (IQR)	0.01 (0.005–0.02)	0.015 (0.009–0.03)	0.012
Survivin -ΔΕx3 (copies/μl), median (IQR)	0.0125 (0.004–0.078)	0.035 (0.007–0.13)	0.250
Survivin WT (copies/μl), median (IQR)	0.007 (0.0017–0.03)	0.022 (0.0035–0.29)	0.019
Survivin -3B (copies/μl), median (IQR)	0.125 (0.01–0.3)	0.16 (0.036–0.35)	0.212
Survivin protein (pg/ml), median (IQR)	134.6 (72.66–227.23)	179.2 (127.65–430.3)	0.001
Caspase -3 (ng/ml), mean ± SE	27.6 ± 4.3	43.1 ± 11.9	0.200
Caspase -9 (ng/ml), mean ± SE	103.5 ± 10.6	129.7 ± 20.9	0.100

*WT* wild type, *SIRS* systematic inflammatory response syndrome.

## Discussion

The complex clinical condition of sepsis may be best defined as the detrimental immunological host response to infection, leading to increased apoptosis of lymphoid and parenchymal tissues, immune suppression, and organ dysfunction^3,4^. Caspases may hold key-roles in sepsis in terms of apoptosis, pyroptosis, necroptosis, and inflammation^6,7,19^. In the present study, we showed for the first time that upregulated total apoptotic caspases in septic patients, including active and cleaved forms, are followed by anti-apoptotic hyperexpression, indicated by increased survivin serum levels, along with increased intracellular survivin transcript variants concentrations in early-onset sepsis. Secondly, we showed that survivin is correlated with IL-8 and caspase-9, which is independently associated with sepsis, achieving the best, among others, sepsis discrimination and outcome predictive-ability. Also, we recorded increased survivin serum protein levels, along with survivin-WT, -2B, and -ΔΕx3 gene expression, among critically ill non-survivors.

Different studies propose that during viral infections, pyroptosis is induced by activation of caspases -3 and -9, through the intrinsic (mitochondrial) apoptotic pathway, whereas survivin levels are being found upregulated or downregulated, amongst a variety of viral strains isolated^10^. Previous studies in mouse brain astrocyte cultures infected with a Picorna-virus strain, revealed the upregulation of the survivin protein in infected cells, confirmed by RT-PCR and qPCR, whereas no enzymatic activity of caspase-3 was detected, due to the formation of survivin/caspase-3 complexes halting the apoptotic process^20^. On the other hand, pilot studies in sepsis caused by bacterial pathogens, such as *Pseudomonas aeruginosa,* have indicated that two types of cells, lymphocytes, and gastrointestinal epithelial cells, undergo accelerated apoptosis^21^.

Apoptosis acts as a down-regulator effector of immune responses in critically ill patients^22^, influencing the severity of systemic response^23^, by declining host’s CD14/HLA-DR expression^24^. The elevated concentrations of total (pro-caspases, active, and cleaved) caspases -3 and -9 among septic patients in this study, might indicate that both the extrinsic and intrinsic pathways are involved in sepsis-induced lymphocyte apoptosis^8^. Besides this, local thymic caspase-9 inhibition decreased lymphocyte apoptosis during polymicrobial sepsis in septic animal models^25^. Similarly, higher caspase-3 activity has been found in lymphocytes of septic patients^26^, involved in brain hippocampal apoptosis in septic animal models^27^. An association of caspase-3 concentrations with sepsis severity, degree of apoptosis, and mortality in septic patients has recently been reported^28^. A caspase-dependent programmed neutrophil death (pyroptosis) associated with inflammation in sepsis is initiated by inflammasomes in innate immunity^29^. In experimental sepsis, LPS resulted in a 1.8-fold increase in myocardial caspase-3 activation and a 6.8-fold increase in apoptotic cardiomyocytes through the Akt/eNOS/NO pathway^30^, whereas cytosolic caspase activation through interferon-β signaling mediated immune responses and lethality^27^. Other experimental studies have also announced that sepsis is accompanied by apoptosis of white blood cells and mainly of CD4 lymphocytes, as a result of both the intrinsic and the extrinsic caspase activation pathways^31^, but monocytes seem to be differently modulated and probably remain unaffected by apoptotic factors in serum^32^.

The IAP family inhibits apoptosis by binding to specific caspases and by other mechanisms, possibly having a central role in the regulation of cellular and intracellular signal transduction^33,34^. The survivin wild-type (full-length/ WT) transcript is a component of chromosome passage protein complex (CPC), composed of at least a triple-helix bundle-based subcomplex with BIRC5/survivin, CDCA8/borealin, and INCENP, AURKB or AURKC, which is essential for chromosome alignment and segregation during mitosis and cytokinesis and may counteract a default induction of apoptosis in G2/M phase preventing apoptosis^35^. Survivin-WT is essential for the maintenance of mitochondrial integrity and function^36^, inhibiting caspases-3 and -7^37^. The increased survivin-WT gene and protein, shown in this study, might be explained by the fact that this isoform can detect the monomer in the CPC-bound state, which efficiently protects cells against apoptosis, as well as the homodimer in the apostate, capable of enhancing tubulin stability in cells. While the monomeric form interacts with cofactor molecules such as the X-linked mammalian IAP protein (XIAP), in order to repress active caspase-9 within the apoptosome^10^, both the dimeric and monomeric form can interact with Smac/DIABLO, which is one of the major antagonists of survivin and XIAP proteins. Accordingly, the simultaneously upregulated caspases and survivin, though not yet fully understood, might indicate an interplay of apoptotic, anti-apoptotic proteins, and cofactors in sepsis^38^. When phosphorylated, survivin has been found to interact with a co-factor, such as HBXIP, in order to exert its anti-apoptotic properties. The resulting complex is then able to bind pro-caspase-9, as well as active caspase-9, although less efficiently^39^.

In addition to the survivin-WT transcript, several transcript variants, generated by alternative splicing of the human survivin gene (BIRC5), have been identified: survivin-ΔΕx3 (a splice variant lacking exon 3), -2B (one variant retaining a part of intron 2 as a cryptic exon), and-3B (a novel exon 3B derived from a portion of intron 3) are currently well studied^40^, whereas other isoforms are not yet satisfactorily characterized^41^. Mokuda et al. have already investigated the expression and function of survivin in different autoimmune diseases, and they have also identified elevated protein expression levels of survivin splice variants in rheumatoid arthritis tissues, confirmed by specific antibodies against survivin’s different splice variants^42,43^. To our knowledge, no study has ever addressed the combined protein/mRNA expression of survivin-WT or the cell-specific expression of other survivin splice variants in septic patients. Several different types of cancers have been shown to express the survivin-WT, -ΔEx3, -2B, -3B splice variants, with no expression in the adjoining normal tissues^44^. Also, serum levels of survivin-WT in active rheumatoid arthritis patients and healthy controls were similar in CD4 + and CD19 + cells, while survivin-2B and -ΔEx3 were significantly higher in CD19 + B cells^45^. In the present study, new forward and reverse primers for each variant were designed to prevent non-specific amplifications, while survivin-WT and -ΔΕx3 were the dominant variants. The expression levels and subcellular localization patterns of each isoform are associated with different functional properties, mainly studied in cancer patients. Survivin-WT, -ΔΕx3, and -3B splice variants seem to have antiapoptotic properties, whereas other studies in autoimmune diseases question the antiapoptotic potential of survivin-3B, and more importantly of survivin-2B and -2α variants. Also, survivin-2B presents its proapoptotic functions by dimerizing with the wild-type, thus reducing its antiapoptotic effects^9,46^.

The survivin protein is expressed during development, its gene encodement contains a BIR domain necessary for its anti-apoptotic functions, and is not expressed in most differentiated adult tissues^47,48^. The overexpression of WT isoform is common in almost all tumors and is indicative of decreased overall survival, increased rate of recurrence, and resistance to therapy^49^. It is the first time that survivin-WT gene or protein expression, as well as total caspases, including active and cleaved forms, are shown to be overexpressed in sepsis and that survivin-ΔEx3, -WT, along with caspase-9 and survivin protein, could better discriminate sepsis among critically ill patients. This finding further enhances results of recent studies, showing that biomolecules, related to repressed bioenergetics and innate immunity^50^, hypo-metabolism and acute hormonal stress^51^, could better discriminate sepsis from SIRS, compared to CRP or lactate.

One possible explanation for survivin higher levels in sepsis could be that the cell is producing as much of antiapoptotic molecules as possible, in an effort to survive. It has been previously shown that, in a dangerous state, protective heat shock proteins are released from damaged cells as “danger signals” activating the host innate and adaptive immune and hormonal response^52,53^. Although the “danger hypothesis” might also explain a simultaneous gene and protein survivin expression upregulation among non-survivors, large-scale trials in the future should focus on multiple downstream pathways concurrently, to further delineate the complex regulatory balance between the different isoforms of survivin, which might determine the response to proapoptotic stimuli^54,55^. Further analyses will also be needed to delineate if the association of total caspases-3 and -9 with sepsis or mortality, might actually reflect an active or cleaved form induction. Assessing a “lymphocyte apoptosis model”, higher lymphocyte apoptotic percentages and lower HLA-DR expression independently predicted mortality in septic patients^56^. Moving the sepsis field onwards, this multicenter study’s findings could be taken into consideration and open up the way for future research on apoptotic/antiapoptotic biomarkers implicated in sepsis outcome.

Several limitations, inherent to our hypothesis-generating/exploratory approach, should be mentioned. Although in our previous studies we separately measured intracellular biomarkers in various cell types (lymphocytes, neutrophils) by flow cytometry, in this “start-up survivin in sepsis” study we only wanted to compare the early-onset antiapoptotic response of survivin to the cumulative extracellular and intracellular apoptotic induction of key caspases-3 and -9 in sepsis. Further studies will be needed to clarify our preliminary survivin-WT gene and protein expression findings, to expand real-time PCR findings of the survivin isoforms produced by alternative splicing at the protein level, to reveal activities of caspases, and, possibly, to detect their relative roles in apoptosis pathways. Although it is possible that the increased levels of caspases in our study might mainly represent the active/cleaved forms of proteins, a series of further studies will certainly be needed to reveal the actual activities of caspases and expand the results of this study. Also, the sepsis sample size did not allow performing robust sub-analyses of specific infectious categories in this study, so that the effort to further expand this process might demand a careful preparation of a larger multi-centered study, in order to elucidate important pyroptotic, apoptotic and antiapoptotic pathways that are activated during the septic cascade. A strength of the study is that this the first time that the antiapoptotic protein survivin is assessed in sepsis. No study has ever studied survivin in septic patients. Since survivin, similar to all other IAPs except XIAP, does not directly bind caspases^38^, we are now in progress of serial blood measurements and more focused analyses in different cell populations, longitudinally studying the complex of activity of intracellularly involved apoptotic (caspases) and anti-apoptotic (survivin, XIAP, Smac/DIABLO) molecules, including more experiments using caspase substrates and antibodies against survivin splice variants (isoforms 2 (2B), 3 (ΔEx3), and 4 (3B)).

In conclusion, the results of this study showed upregulation of caspases-3, -9, and survivin serum proteins, along with an escalated increase of survivin-WT, -2B, -ΔΕx3 mRNA, and repressed survivin-3B mRNA in early-onset sepsis. Possible influences of simultaneously increased survivin-WT gene expression and protein, along with the produced by alternative splicing survivin-2B and -ΔΕx3 gene expressions on outcome, are also reported for the first time. A presumed key-role of caspase-9 in septic cascades should be urgently delineated. Any possible association of apoptotic status with organ failure and mortality might be clinically important, insinuating a critical role in the pathogenesis and development of sepsis and septic shock with potentially important future scientific implications.

## Materials and methods

### Study design

In this prospective, cross-sectional, observational study, 4 ICUs were recruited. All methods were carried out in accordance with relevant guidelines and regulations that comply with institutional, national and international guidelines. The study was approved by the local ethics committee (University Hospital of Heraklion Institutional Review Board; Approval No. 9452/26.6.16) and was performed during a 48-month period, between 2017 and 2019. Written informed consent was obtained from a first-degree relative of patients admitted to the ICUs.

### Patients

Adult patients (> 18 years-old) consecutively admitted with early-onset (< 24 h) sepsis/septic shock or traumatic SIRS were eligible for enrolment. The sepsis group included patients with an identified source of infection and Sequential Organ Failure Assessment (SOFA) score > 2^57^, according to the updated Sepsis-3 definition for adults, while the septic shock criteria (need for vasopressor support to maintain MAP > 65 mmHg and lactate levels > 2 mmol/L) were used to identify patients with septic shock^58^. The non-infectious SIRS group included trauma patients who met at least two of the four conventional criteria for SIRS^59^ and represented the first control group (ICU control). Healthy volunteers represented the second control group (healthy individuals). Exclusion criteria were malignancy, immune deficiency and late-sepsis or SIRS > 24 h after admission.

### Clinical characteristics

Demographic data, clinical details and patients’ outcome hardpoints were recorded through patients’ medical charts. The primary clinical outcomes were mortality and severity of illness, assessed through standardized severity scoring systems on admission. The Acute Physiology and Chronic Evaluation-II (APACHE II), Multiple Organ Dysfunction (MODS), Simplified Acute Physiology Score-III (SAPS III), and SOFA score^57^ were recorded on admission. ICU length of stay (LOS) and multiple inflammatory indices were assessed as secondary clinical outcomes. For this purpose, serum levels of lactate, CRP and various organ function parameters were also recorded.

### Assays

Data have been inconsistent regarding survivin isoforms, their cell type expressions or interphase ratios and, since this is a first trial in sepsis^34,60^, survivin was measured in white blood cells, in order to present its total intracellular potential, and in serum as well, so as to assess the comparative extracellular concentrations of the actively secreted survivin, the way other biomolecules and caspases have been previously studied^61–66^.

Blood samples were obtained within 24 h of admission to the ICU and then stored at −80 °C until total RNA extraction. The intracellular cumulative transcriptional mRNA expression of survivin in white blood cells was assessed through real-time quantitative polymerase chain reaction (qPCR), which was performed on the complementary DNA (cDNA), using reverse primers and hybridization probes, specific for the genes of interest for each splice variant of survivin (RNA extraction and reverse transcription for cDNA synthesis). New forward and reverse primers for each variant were designed to prevent non-specific amplifications (Appendix Table C1 of forward and reverse primers for each survivin variant). An illustration of primer location in Exons 1- 4 is provided in Supplementary Figure S1.

Total mRNA was isolated from blood samples and K652 human cell line, using the Trizol reagent (Monarch Total RNA Miniprep Kit, NEB #T2010, New England Biolabs, UK) and reverse transcription was performed using an iScript cDNA synthesis kit (BioRad, 1708891, Hercules, CA, USA) according to the manufacturer’s instructions. qRT-PCR analysis was carried out using iTaq SYBR Green Universal Supermix (BioRad, 1725122, Hercules, CA USA) in a Thermal Cycler real time PCR detection system (BioRad CFX96, USA). Melting curve experiments had previously established that the fluorescence signal for each amplicon was derived from the products only, and no primer dimers were found (Supplementary Figure S2).

Absolute quantification was applied by calculating the ratio of the number of the molecules (copies/μl) of the target gene to the number of the molecules (copies/μl) of the reference gene (β-actin). In comparison to the relative fluorescence intensity changes (qualitative method), cDNA based absolute qPCR quantification is found to be more sensitive to gene expression variations caused by factors such as environmental variations^67^. The internal standards were not based in cDNA library but consisted of tenfold serial dilutions of K562 cell line cDNA. The real-time PCR efficiencies were calculated from the slope. Amplification efficiency was similar (1684–1986) between the target and the reference gene respectively. RNA samples from 182 pathological and 89 healthy blood samples were calculated, providing a fine comparison. The evaluation for the proper size and purity of PCR samples was performed through electrophoresis on agarose gels (Supplementary Figure S3).

Protein serum levels of survivin (eLabscience, E-EL-H1584, USA), along with caspase-3 (eLabscience, E-EL-H0017, USA) and -9 (eLabscience, E-EL-H0663, USA) were measured extracellularly through quantification analysis, with an enzyme-linked immunosorbent assay (ELISA) method and according to the manufacturer’s instructions. The E-EL-H0017 and E-EL-H0663 kits detect pro-caspase-3, the cleaved subunits, and the active heterodimer formed by p17 and p12 subunits, and are suitable for testing cleaved caspase-3 and -9, respectively (eLabscience Biotechnology, Houston). The E-EL-H1584 detects survivin-wild-type protein (isoform 1, Baculoviral IAP repeat-containing protein 5, length 142, also known as alpha, apoptosis inhibitor 4, or apoptosis inhibitor survivin) (UniParc, identifier: O15392-1), chosen as the canonical sequence (eLabscience Biotechnology, Houston). Accordingly, survivin protein in this study only represents the isoform 1 (WT). The sequence of 2B differs from the canonical sequence as follows: 74-74aa: I → IGPGTVAYACNTSTLGGRGGRITR. Obviously, the sequence of the regions is different between isoform 1 and isoform 2 and the length of the two isoforms are also different. All specimens were assayed twice. The detection range was around 1.58 to 9.96 pg/ml for survivin, 0.31–20 ng/mL for caspase-3 and 1.563–100 ng/mL for caspase-9.

The patients’ cytokine profile (interleukins (IL) -6, -8, -10) was also evaluated using a commercially available ELISA kit (Invitrogen, Carlsbad, CA, USA). The sensitivities of the assays were < 2 pg/ml for IL-6, < 2 pg/ml for IL-8 and < 1 pg/ml for IL-10. As far as routine laboratory measurements are concerned, CRP assays were performed on Beckman Coulter AU Analyzers (Beckman Coulter, AU US). CRP levels > 0.8 mg/dl were considered abnormal. PCT was measured by a latex enhanced immunoturbinometric assay (Diazyme- PCT Assay, USA). Detection threshold was 0.1 ng/ml; PCT levels > 0.5 ng/ml were considered abnormal (laboratory cut off values).

### Statistical analyses

To calculate an adequate sample size, we used the G*Power statistical power calculator. Statistical test ANOVA (F-test): Fixed effects special, main effects and interactions; power = 0.80, alpha = 0.05, effect size medium (f = 0.25). The calculated total sample size was 200 (all groups), critical F = 2.42, non-centrality parameter λ = 12.5, denominator df = 195. Levene’s test of the homogeneity of group variances was used to determine the data distribution from measured variables. Categorical variables are described in absolute values and frequency. Quantitative variables are expressed in mean and standard deviation (if symmetrical) or, in median and interquartile range (if asymmetrical). ANOVA with Tukey post hoc tests or the Kruskal–Wallis independent samples test with multiple comparison analyses, using post hoc Dunn’s pairwise tests with Bonferroni corrections, were used to perform comparisons among parametric or nonparametric groups, as appropriate. Between-group comparisons were conducted using the χ^2^ test for categorical parameters and Spearman’s correlation coefficient for correlation between two continuous variables. To keep the demographic data homogeneity between sepsis and control groups, data was assessed using a propensity score analysis, by the nearest neighbor matching, with a caliper of 0.2SD of the logit of the propensity score. In the well-matched groups, a logistic regression model (backward likelihood ratio) was adopted to examine whether any of the studied variables are independent discriminators of sepsis. We first used univariate models to test all clinical and laboratory variables related to sepsis with just one explanatory variable at a time; afterwards, all variables that had shown a relaxed *p* value of less than or equal to 0.1 were included in the multivariate model. To evaluate discriminative values, the areas under the receiver operating characteristic curves (AUROCs) for variables significantly differing between sepsis and non-sepsis groups and between survivors and non-survivors were calculated. The Youden J index to select the prognostic cut-off value for each studied biomolecule was used. Statistical analysis software (version 25, SPSS, Chicago, IL) was used for all analyses.

## Supplementary information

Supplementary Information.

## Data Availability

The different institutions’ rights, including legal and ethical concerns, patient privacy, and confidentiality restrict access to this multicenter study data sharing.
