# Supplementary material for: Survivin and caspases serum protein levels and survivin variants mRNA expression in sepsis
Source: Sci Rep. 2021 Jan 13;11:1049. doi: 10.1038/s41598-020-78208-2 (PMC7806640; doi:10.1038/s41598-020-78208-2)

**TITLE PAGE**

**Survivin and caspases serum protein levels and survivin variants mRNA expression in sepsis**

*Running title: Survivin and caspases in in early-onset sepsis*

Marianna Miliaraki, MD, MSc^1^, Panagiotis Briassoulis, MD, PhD^1^, Stavroula Ilia MD, PhD^1^, Aikaterini Polonifi, PhD^2^, Marina Mantzourani, MD, PhD^2^, Efrossini Briassouli, MD, PhD^2^, Konstantinos Vardas, MD, PhD^3^, Serafim Nanas, MD, PhD^3^, Aikaterini Pistiki, PhD^4^, Maria Theodorakopoulou, MD, PhD^5^, Theonymfi Tavladaki, MD, PhD^1^, Anna Maria Spanaki, MD, PhD^1^, Eumorfia Kondili, MD, PhD^6^, Helen Dimitriou, PhD^7^, Sotirios Tsiodras, MD, PhD^4^, Dimitrios Georgopoulos, MD, PhD^6^, Apostolos Armaganidis, MD, PhD^5^, George Daikos, MD, PhD^2^, George Briassoulis, MD, PhD*^,1^

*^1^Pediatric Intensive Care Unit, Medical School, University of Crete, Heraklion, Crete, Greece*

*^2^First Department of Internal Medicine - Propaedeutic, National and Kapodistrian University of Athens, Greece*

*^3^First Critical Care Department, Evangelismos University Hospital, National and Kapodistrian University of Athens, Greece*

*^4^4^th^ Department of Internal Medicine, Attikon University Hospital, National and Kapodistrian University of Athens, Greece*

*^5^ 2^nd^ Department of Critical Care, Attikon University Hospital, Medical School, National and Kapodistrian University of Athens, Athens, Greece*

*^6^ Intensive Care Unit, Medical School, University of Crete, Heraklion, Crete, Greece*

*^7^ Division of Mother and Child Health, Medical School, University of Crete, Heraklion, Crete, Greece*

Financial disclosure(s): None declared

Conflicts of interest: None declared

* Correspondence: George Briassoulis, MD, PhD

Professor*_em_*, Medical School, Postgraduate program “Emergencies and Intensive Care in Children Adolescents and Young Adults”, University of Crete

71500 Voutes, Heraklion, Crete, GREECE

Tel.: +30-2810-394675, Fax: +30-2810-392652;

E-mail: [ggbriass@otenet.gr](mailto:ggbriass@otenet.gr); ORCID ID: 0000-0003-0928-6183;

Additional e-mails:

1) marianmyl@yahoo.gr; ORCID ID: 0000-0001-7622-202X;

2) stavroula.ilia@uoc.gr; ORCID ID: 0000-0002-0936-368X

3) [briaspan@hotmail.com](mailto:briaspan@hotmail.com); ORCID ID: 0000-0002-9789-9002


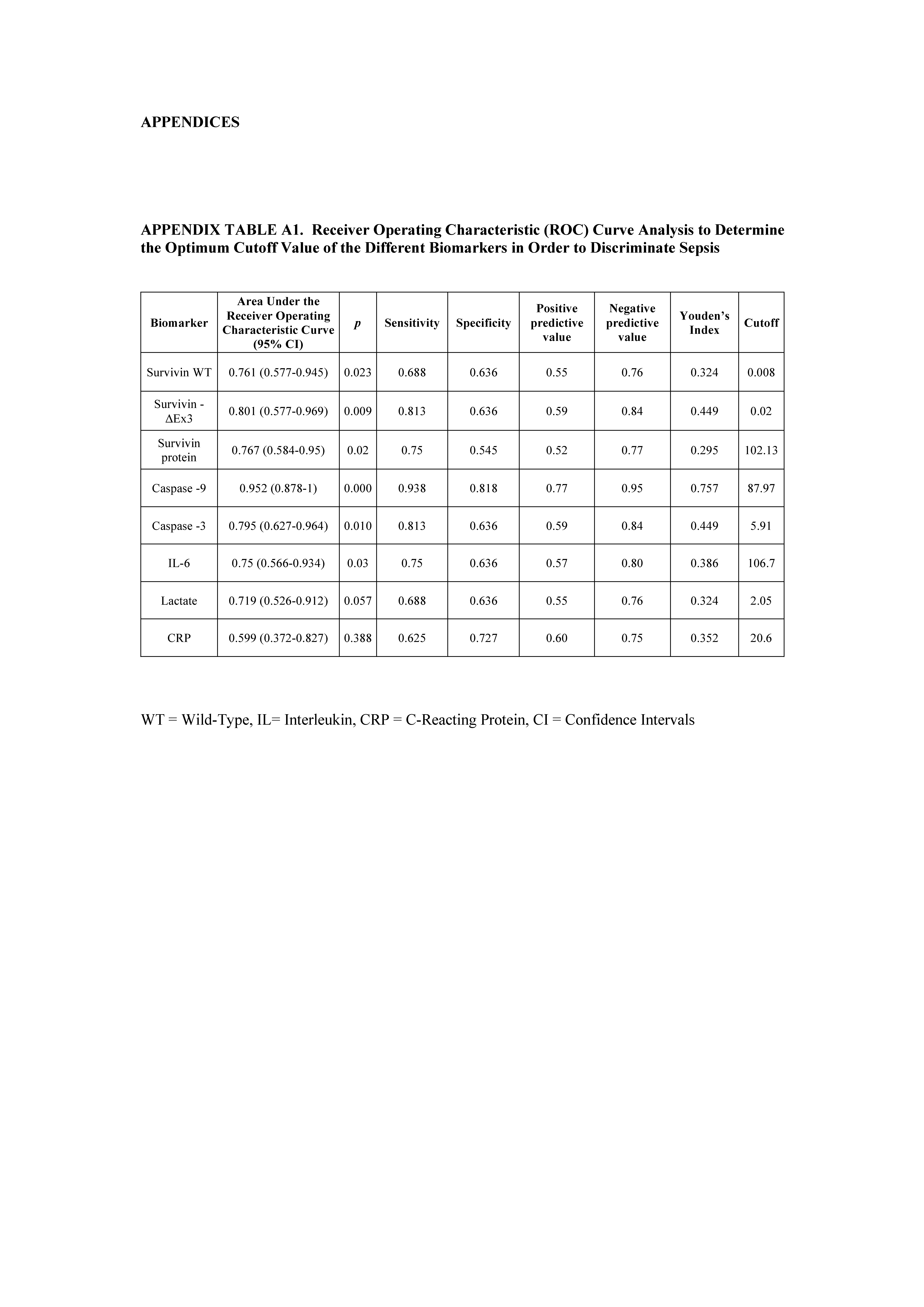


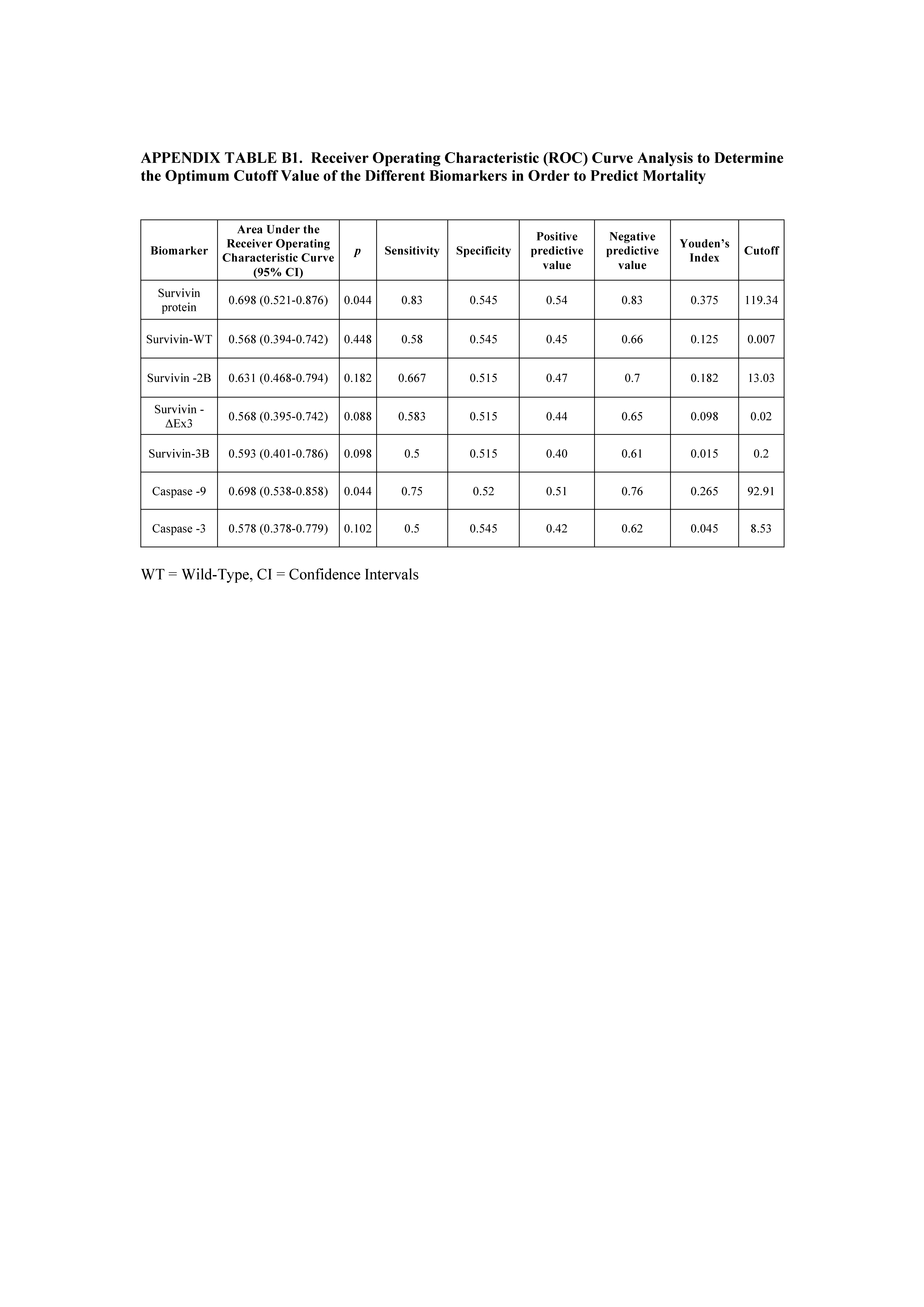


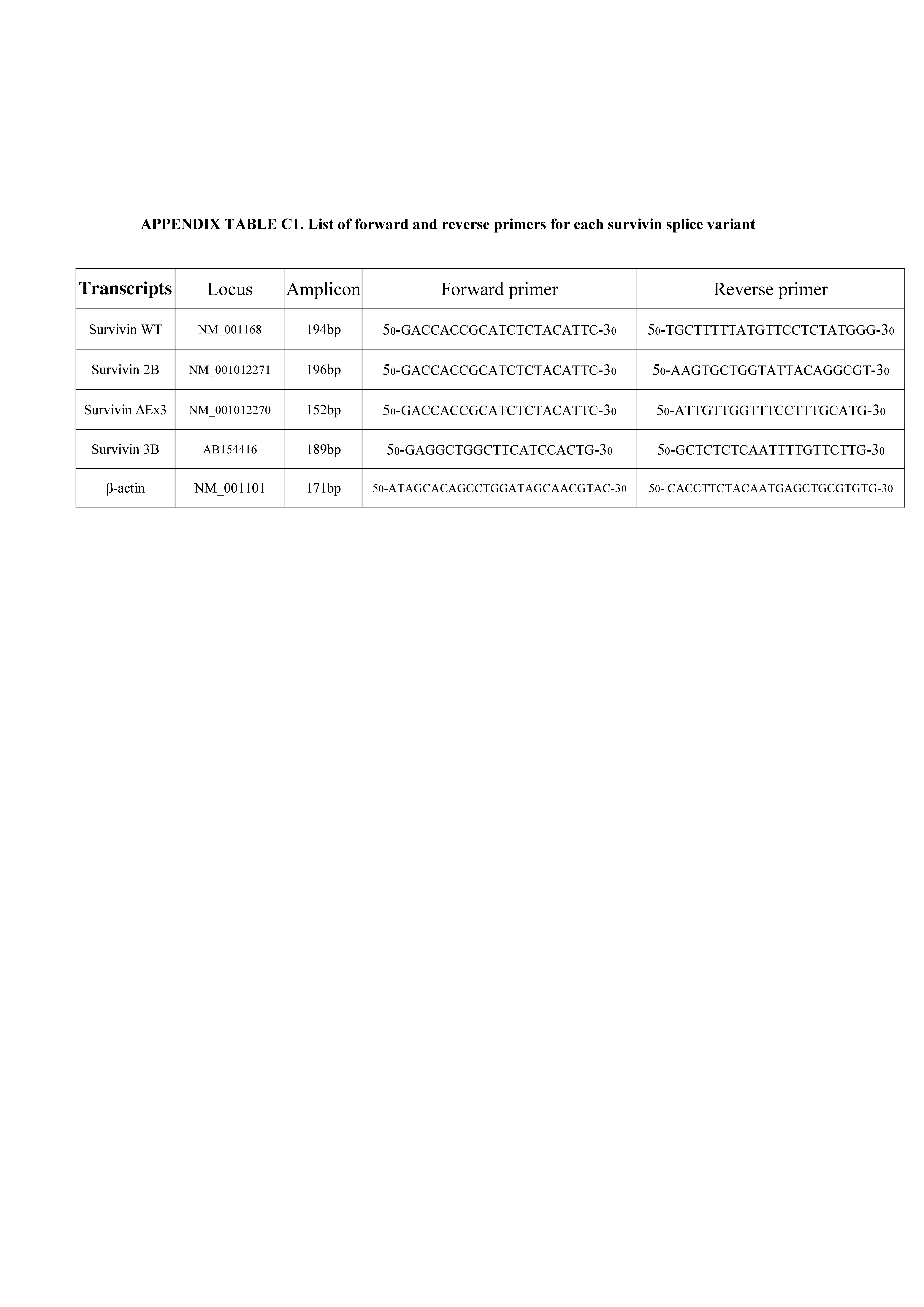


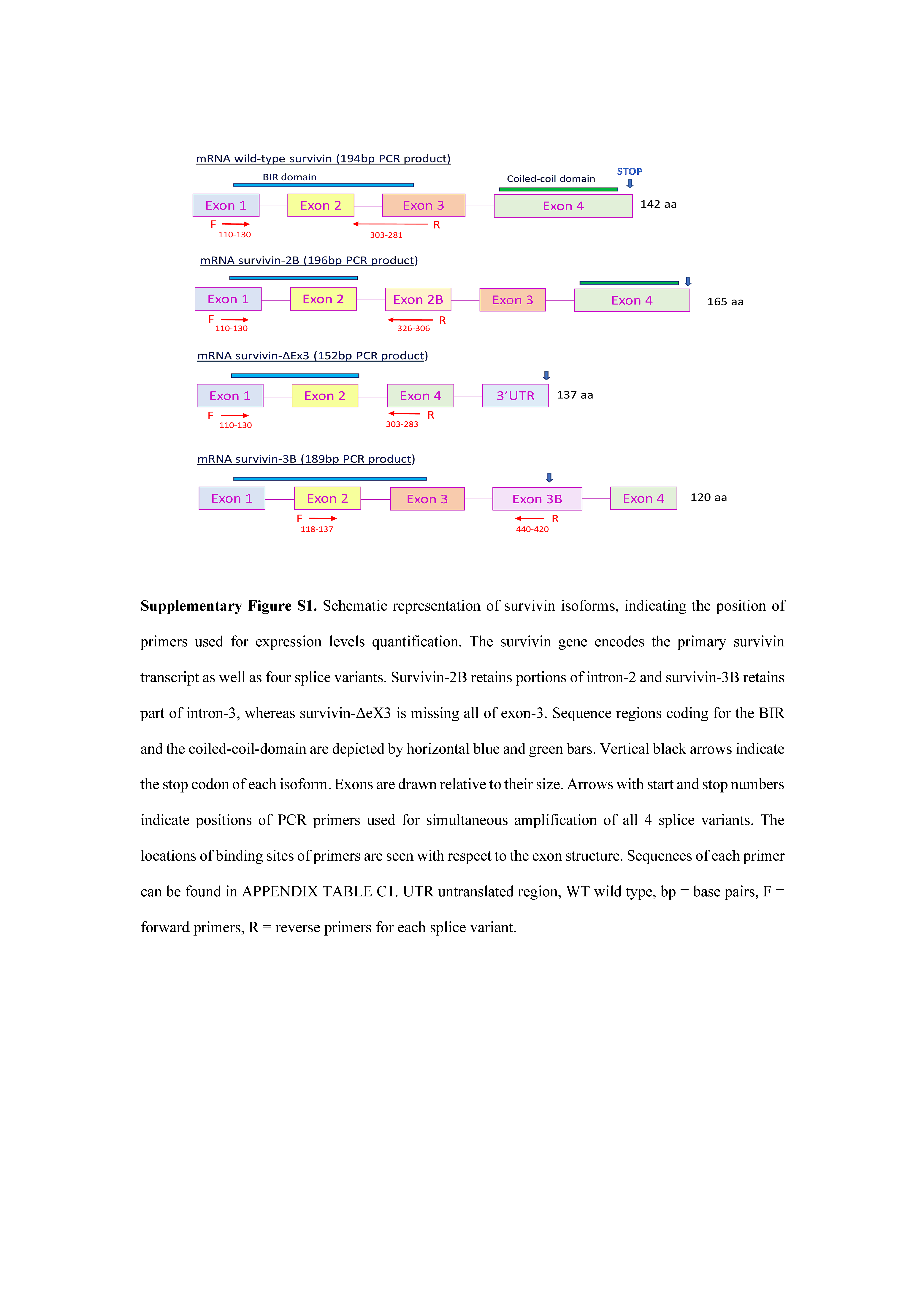


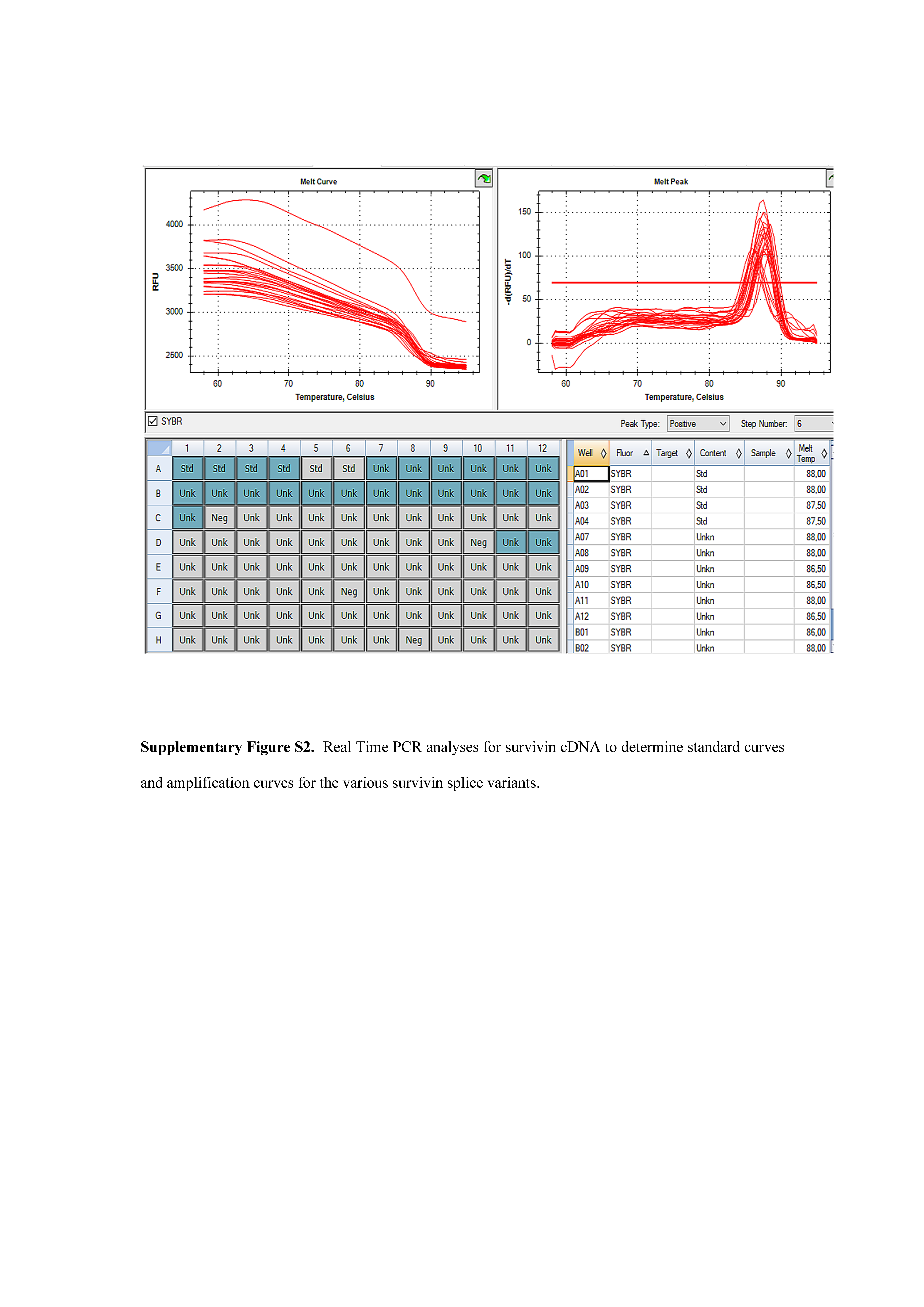


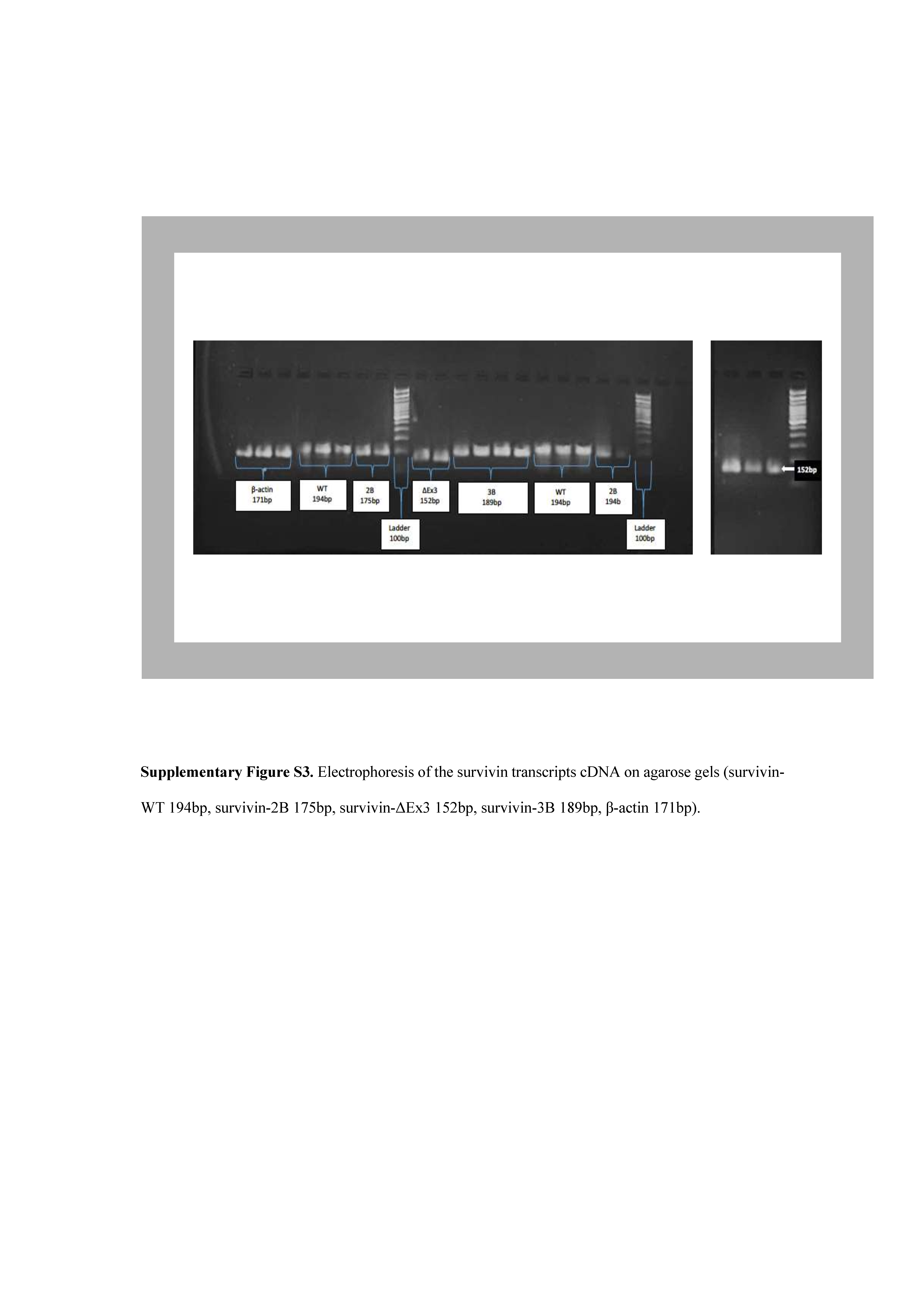

Supplement: Supplementary file 1 — Supplementary Information. [file 41598_2020_78208_MOESM1_ESM.docx]
